# KMnO_4_/Pb staining allows uranium free imaging of tissue architectures in low vacuum scanning electron microscopy

**DOI:** 10.1038/s44303-024-00045-z

**Published:** 2024-10-01

**Authors:** Akira Sawaguchi, Takeshi Kamimura, Kyoko Kitagawa, Yoko Nagashima, Nobuyasu Takahashi

**Affiliations:** 1 Division of Ultrastructural Cell Biology, Department of Anatomy, Faculty of Medicine, University of Miyazaki, Miyazaki, 889-1692 Japan; 2 Hitachi High-Tech Corporation, Tokyo, 105-6409 Japan

**Keywords:** Histology, Microscopy, Scanning electron microscopy

## Abstract

Scanning electron microscopy under low-vacuum conditions allows high-resolution imaging of complex cell/tissue architectures in nonconductive specimens. However, the conventional methods for metal staining of biological specimens require harmful uranium compounds, which hampers the applications of electron microscopy. Here, we introduce a uranium-free KMnO_4_/Pb metal staining protocol that allows multiscale imaging of extensive cell/tissue architectures to intensive subcellular ultrastructures. The obtained image contrast was equivalent to that of Ur/Pb staining and sufficient for ultrastructural observation, showing the fine processes of podocytes in the glomerulus, which were invisible by light microscopy. The stainability in the elastic tissue indicated that the distinct histochemical properties of KMnO_4_ oxidation led to Pb deposition and BSE signal enhancement superior to Ur staining. Elemental analysis clarified that the determinant of the backscattered electron signal intensity was the amount of Pb deposition enhanced by KMnO_4_ oxidation. This user-friendly method is anticipated to create a new approach for biomedical electron microscopy.

## Introduction

One of the major goals of bio-imaging is to elucidate the correlations between cell/tissue structure and function. Electron microscopy is well established as one of the most powerful tools for the study of ultrastructures^1,2^. In the last decade, many cell biologists have used immunocytochemistry benefiting from super-resolution fluorescence microscopy, but the super-resolution microscopy techniques continue to rely on fluorescence labelling as their foundational principle. In this technical context, electron microscopy observations are indispensable approach and still developing with emerging devices/methods to correlate cell/tissue structure and function in biology^3,4^.

Scanning electron microscopy (SEM) provides three-dimensional information on specimen surfaces by collecting the backscattered electrons (BSEs) that are high-energy electrons reflected from the sample by the elastic scattering of the primary beam electrons. Low-vacuum SEM (LvSEM) allows BSE imaging of nonconductive biological samples because the negative charge that accumulates on the nonconductive materials can be neutralised by the positive ions in the residual gas molecules^5–7^. Recent advances in the ultrastructural analysis of nonconductive paraffin sections have exploited low-vacuum conditions, which prevent the accumulation of negative charges^8–11^.

Bridging light and electron microscopy, correlative light and electron microscopy (CLEM)^12^ has been established to combine the molecular specificity of fluorescence microscopy and the high spatial resolution of electron microscopy. Researchers have recently applied CLEM, taking it in the wide sense using conventional light microscopy instead of fluorescence one, to determine the orientation of complex cell/tissue architectures by light microscopy and identify the ultrastructural correlations on the identical paraffin section via LvSEM (PS-LvSEM)^13–15^.

We previously introduced an informative three-dimensional survey of complex cell/tissue architectures in thick paraffin sections by low-vacuum scanning electron microscopy (Thick PS-LvSEM)^10^. However, the conventional metal staining methods used to visualise the cell/tissue architectures require harmful uranium compounds^16^, which hampers the universal application of CLEM imaging by PS-LvSEM and three-dimensional imaging by Thick PS-LvSEM. To date, several reports have introduced compounds as substitutes for Ur/Pb metal staining, such as platinum blue (Pt-blue)^17^, oolong tea extract^18^, samarium triacetate^19^, gadolinium triacetate^19^ or lanthanide salts^19,20^, but these compounds are originally introduced for transmission electron microscopy of ultrathin sections. To the best of our knowledge, only Pt-blue staining has been applied to paraffin sections for differential enhancement of the BSE signal from epithelial tissue, endothelium and mast cells in LvSEM^21^.

The aim of this study was to develop a safe and rapid uranium-free metal staining method, which remains challenging due to difficulties in achieving satisfactory BSE signal enhancement. To address this problem, we applied simple potassium permanganate (KMnO_4_) oxidation combined with conventional lead citrate metal staining (KMnO_4_/Pb metal staining). Here, we describe the precise procedures of this new multiscale LvSEM method involving uranium-free KMnO_4_/Pb metal staining for biomedical research.

## Results

### Practical flow of multiscale PS-LvSEM imaging by KMnO_4_/Pb metal staining

**Fig. 1:**
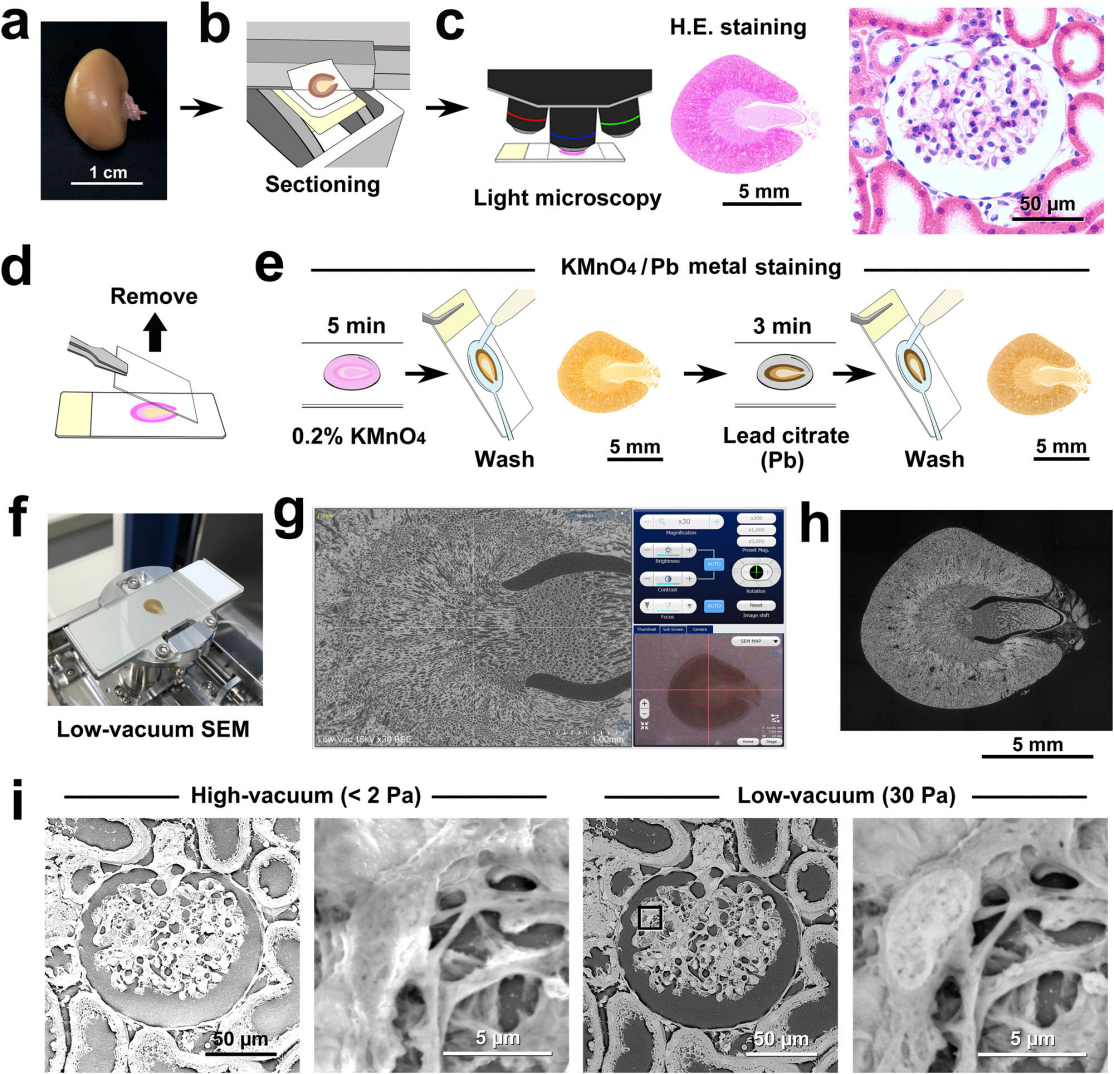
Practical flow diagram of correlative light microscopy and PS-LvSEM imaging by KMnO_4_/Pb metal staining. **a** Gross view of the rat kidney. **b**–**e** Procedure for correlative light and electron microscopy. **b** Generation of paraffin-embedded kidney sections. **c** Light microscopic survey by haematoxylin and eosin staining. **d** Removal of the coverslip. **e** Oxidative treatment with 0.2% KMnO_4_ followed by the application of Reynold’s lead citrate solution. **f** Setting onto the slide-glass holder. **g** The operation screen combined with the camera navigation window (lower right). **h** Montage image of the whole section (compare to the light micrograph in (**c**). **i** Correlative light microscopy and PS-LvSEM images of the renal corpuscle, correlating to the light micrograph in (**c**), without charge-up obstruction in low-vacuum mode at 30 Pa. The high-power view shows the processes of podocytes in the glomerulus.

Figure 1 shows the flow diagram of multiscale PS-LvSEM, from the centimetre-scale light microscopic survey to the nanometre-scale correlative PS-LvSEM imaging of the rat renal corpuscle by KMnO_4_/Pb metal staining. After the light microscopic survey by haematoxylin and eosin (H.E.) staining (Fig. 1c), the sections were treated with 0.2% KMnO_4_ for 5 min, followed by lead citrate for 3 min (Fig. 1e) for correlative PS-LvSEM imaging (Fig. 1f–i). The operation screen combined with the camera navigation window assists in seamless light and electron microscopy observation (Fig. 1g). The semiautomatic capture of tilling PS-LvSEM images (Supplementary Fig. 1) yielded a montage image of the whole section (Fig. 1h). As a result, in contrast to high-vacuum conditions (<2 Pa), LvSEM imaging by KMnO_4_/Pb metal staining clearly showed podocytes in the renal corpuscle without charge-up obstruction in low-vacuum mode at 30 Pa (Fig. 1i). The high-power view revealed the fine processes of podocytes which were invisible under the conventional light microscopic survey by H.E. staining.

For fine structural observation, the physical property of the electron beam required us to tune the acceleration voltage according to the magnification of the target structure (Supplementary Fig. 2) because of the trade-off between observing the surface undulations at 5 kV (low contrast) and not seeing the surface undulations at 20 kV (high contrast).

### Evidence-based coordination of KMnO_4_/Pb metal staining

**Fig. 2:**
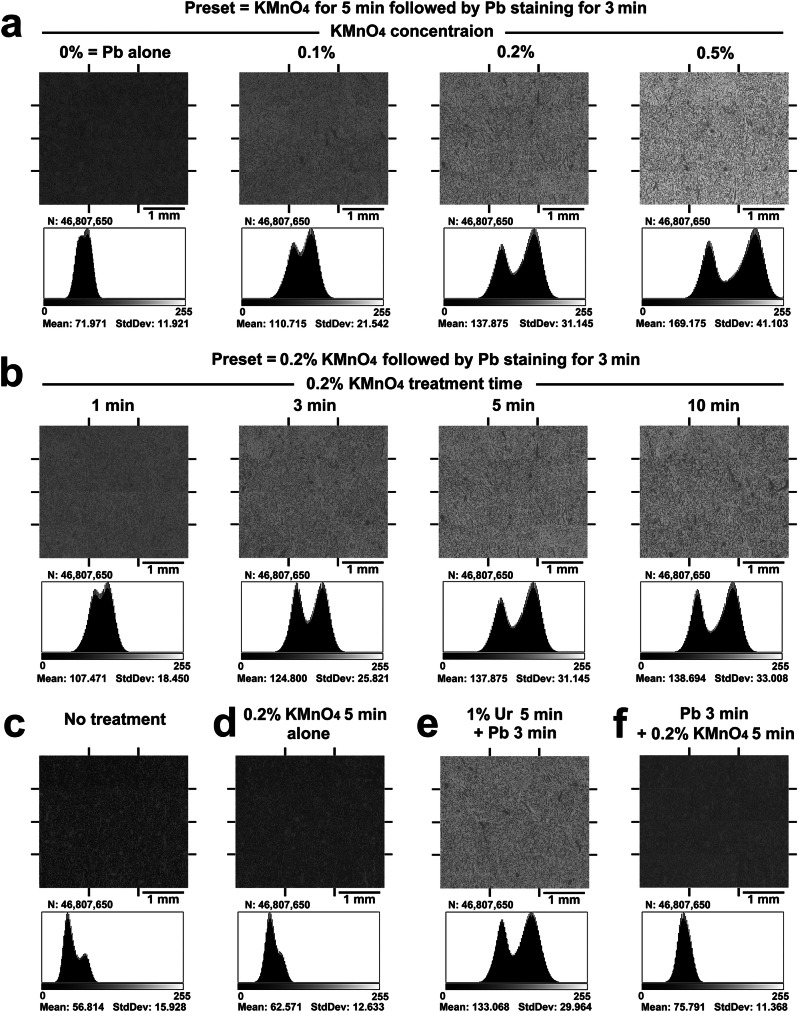
Histogram analysis to determine the optimal KMnO_4_/Pb metal staining protocol. Electron micrographs were randomly collected from the renal cortex and tilled for histogram analysis (Supplementary Fig. 3). Grayscale histograms depicting the number of pixels at each grey level, ranging from 0 to 255, present in each staining protocol. **a** All sections were preset for treatment with KMnO_4_ for 5 min followed by Pb staining for 3 min. **b** All sections were preset for treatment with 0.2% KMnO_4_ followed by Pb staining for 3 min. **c** No treatment; **d** 0.2% KMnO_4_ for 5 min, alone; **e** 1% uranyl acetate for 5 min followed by Pb staining for 3 min; and **f** Pb staining for 3 min followed by 0.2% KMnO_4_ for 5 min. Note the representative histogram after treatment with 0.2% KMnO_4_ for 5 min followed by Pb staining for 3 min (**a**, **b**), corresponding to the conventional treatment with 1% uranyl acetate for 5 min followed by Pb staining for 3 min (**e**). N total number of pixels, Mean mean grey level, StdDev standard deviation.

The optimal protocol for KMnO_4_/Pb metal staining was determined by histogram analysis (Fig. 2) on the electron micrographs randomly collected from the renal cortex and tilled, as shown in Supplementary Fig. 3. Grayscale histograms depicted the number of pixels at each grey level, ranging from 0 to 255, present in each staining protocol. In general, a high contrast image yields the bimodal distribution of statistical plots. As the contrast increases, the two peaks are moved farther apart, and the “valley” between the peaks becomes more pronounced.

**Fig. 3:**
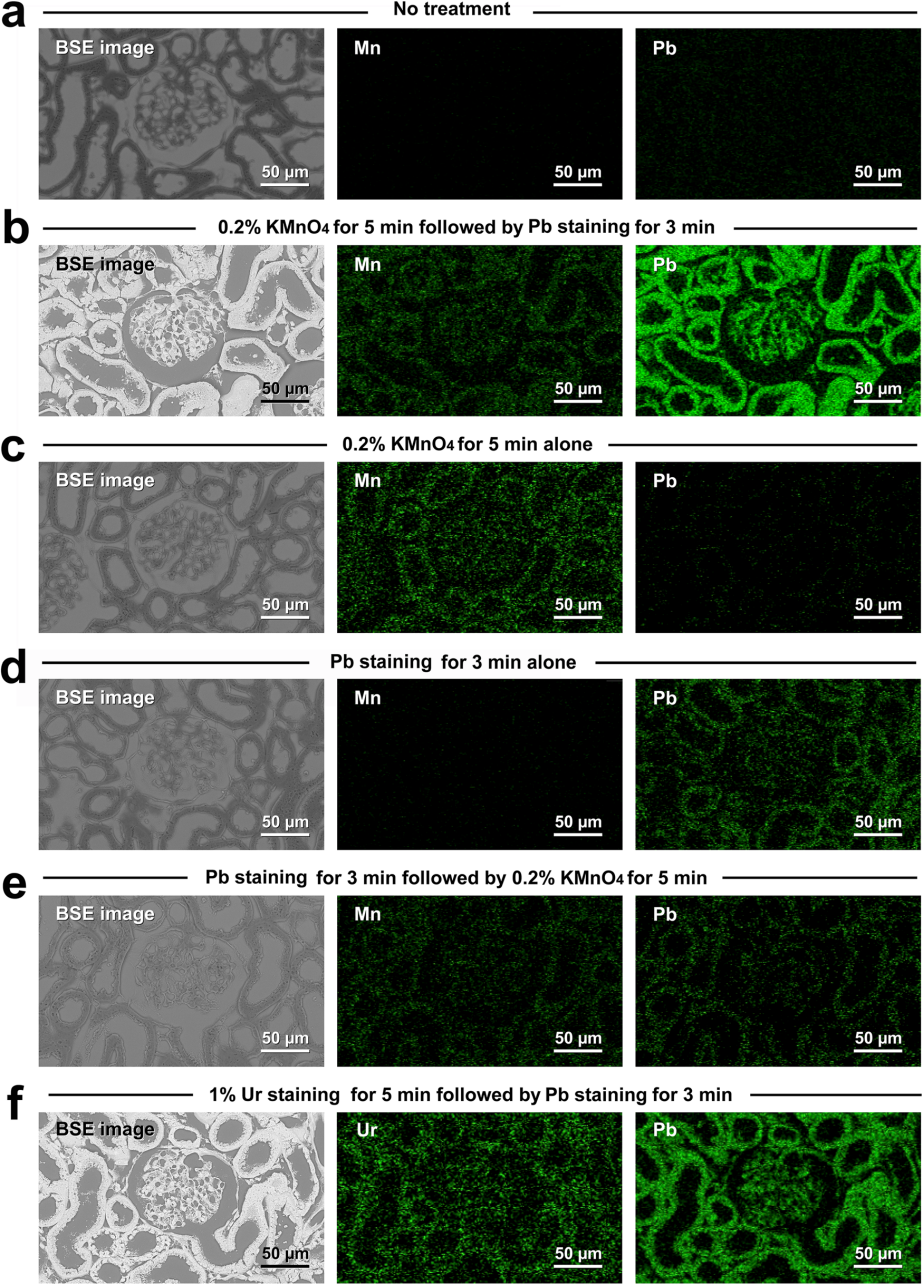
Elemental analysis of the metal distribution after diverse staining protocols. Elemental mapping indicating the distributions of manganese (Mn), lead (Pb) and uranium (Ur) after diverse staining protocols. **a** No treatment; **b** 0.2% KMnO_4_ for 5 min, followed by Pb staining for 3 min; **c** 0.2% KMnO_4_ for 5 min, alone; **d** Pb staining for 3 min, alone; **e** Pb staining for 3 min followed by 0.2% KMnO_4_ for 5 min; and **f** 1% uranyl acetate for 5 min followed by Pb staining for 3 min. Note the intense Pb distribution in the high-contrast PS-LvSEM images in (**b**, **f**), which corresponds to the renal corpuscle and uriniferous tube.

First, the optimal concentration of KMnO_4_ was determined to be in the range of 0.1–0.5% (Fig. 2a) by presetting KMnO_4_ treatment for 5 min followed by Pb metal staining for 3 min. Next, the optimal treatment time was determined to be in the range of 1–10 min for standard treatment with 0.2% KMnO_4_ followed by Pb metal staining for 3 min (Fig. 2b). As a control, it was confirmed that the BSE signal intensity did not increase by oxidation with KMnO_4_ alone (compare Fig. 2c, d). Finally, the optimal protocol was determined to be 0.2% KMnO_4_ for 5 min followed by Pb metal staining for 3 min according to the BSE signal intensity and histogram patterns of conventional Ur/Pb staining (Fig. 2e). Further elemental analysis revealed the distributions of manganese, lead, and uranium (Fig. 3) and revealed that the determinant of BSE signal intensity was the amount of Pb deposition enhanced by KMnO_4_ oxidation (Fig. 3b) and Ur metal staining (Fig. 3f) compared to the control with Pb staining alone (Fig. 3d). It should also be noted that Pb staining must be preceded by KMnO_4_ oxidation to obtain a sufficient BSE signal (Fig. 2f, 3e).

### Competitive evaluation of the optimised KMnO_4_/Pb metal staining procedure versus conventional Ur/Pb metal staining

**Fig. 4:**
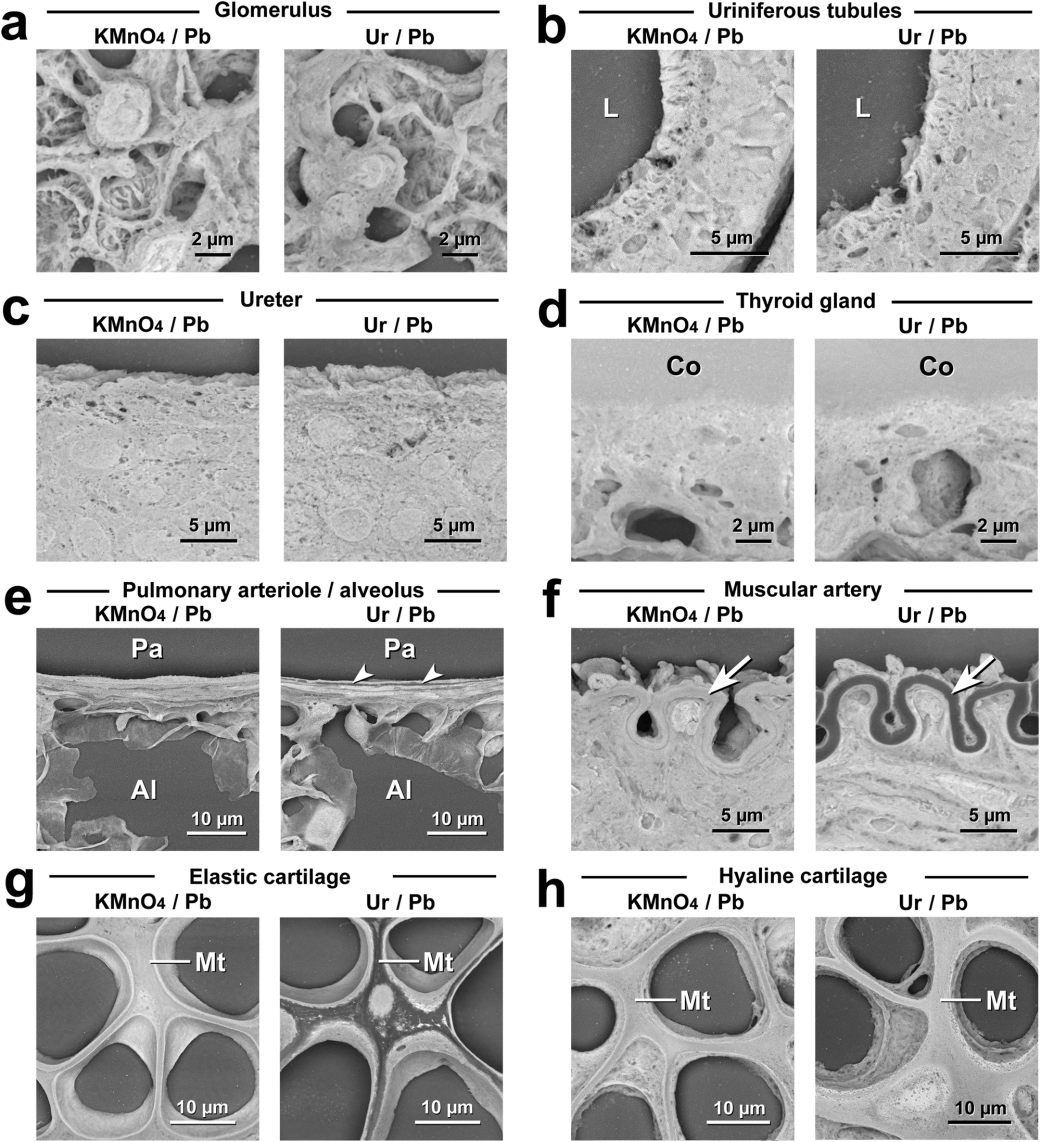
Comparison of PS-LvSEM imaging after KMnO_4_/Pb and Ur/Pb metal staining. **a** Glomerulus in the renal corpuscle. **b** Uriniferous tubules. L lumen. **c** Transitional epithelium in the ureter. **d** Follicular epithelium and colloid (Co) in the thyroid gland. **e** Pulmonary arteriole (Pa) and alveolus (Al). Arrowheads indicate the dark layer by Ur/Pb metal staining. **f** Muscular artery in the kidney. Note the internal elastic lamina (arrows) and their dark appearance after Ur/Pb metal staining. **g** Elastic cartilage in the auricle. Note the interterritorial matrix (Mt) and its dark appearance after Ur/Pb metal staining. **h** Hyaline cartilage in the trachea. Note the bright appearance of the interterritorial matrix (Mt) after both KMnO_4_/Pb and Ur/Pb metal staining.

The optimised protocol for KMnO_4_/Pb metal staining was evaluated for comparison with the conventional Ur/Pb metal staining method (Fig. 4). The stainability and image contrast seemed to be equal in the glomerulus (Fig. 4a), the uriniferous tube (Fig. 4b), the transitional epithelium of the ureter (Fig. 4c), and the follicular epithelium and colloid in the thyroid gland (Fig. 4d). On the other hand, the pulmonary arteriole exhibited dark layers after Ur/Pb metal staining (Fig. 4e). In relation to the elastin-rich structure, conventional Ur/Pb metal staining provided similar dark areas in the internal elastic lamina of the muscular artery (Fig. 4f) and the interterritorial matrix of the elastic cartilage (Fig. 4g) in contrast to KMnO_4_/Pb metal staining. Additional elemental analysis revealed that the deposition of Mn and Pb by KMnO_4_/Pb metal staining resulted in the interterritorial matrix having a bright appearance (Supplementary Fig. 4). In contrast, the interterritorial matrix of the hyaline cartilage exhibited a bright appearance after Ur/Pb metal staining that was equivalent to that after KMnO_4_/Pb metal staining (Fig. 4h).

**Fig. 5:**
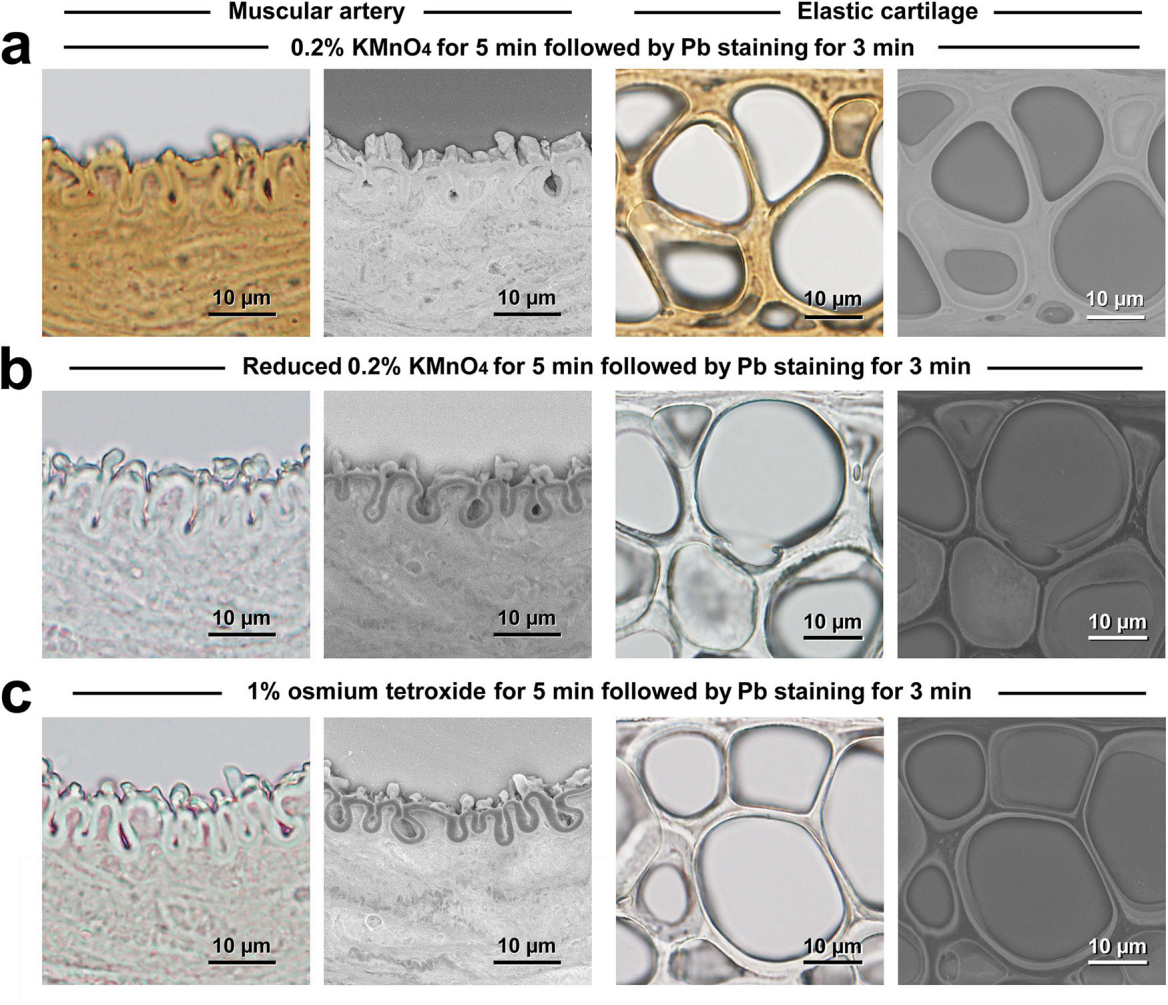
Dependence on oxidation by KMnO_4_ for high-contrast PS-LvSEM imaging. Correlative light microscopy and PS-LvSEM images after treatment with oxidative 0.2% KMnO_4_ for 5 min followed by Pb staining for 3 min (**a**), nonoxidative reduced 0.2% KMnO_4_ for 5 min followed by Pb staining for 3 min (**b**), and oxidative 1% osmium tetroxide for 5 min followed by Pb staining for 3 min (**c**). Note the brownish-light microscopic appearance and the high contrast of the PS-LvSEM image obtained after KMnO_4_/Pb metal staining.

These findings indicated that the distinctive histochemical property of KMnO_4_ oxidation led to Pb deposition and BSE signal enhancement. The importance of oxidation was confirmed by treatment with reduced KMnO_4_, which failed to enhance the BSE signal (Fig. 5a, b). We also examined osmium tetroxide, which is commonly used in electron microscopy as a postfixative oxidising agent, but this treatment resulted in only partial BSE signal enhancement insufficient for fine structural LvSEM imaging (Fig. 5c).

### Comparative LvSEM imaging after KMnO_4_/Pb, Pt-blue/Pb, OTE/Pb, and Sm/Pb metal staining

**Fig. 6:**
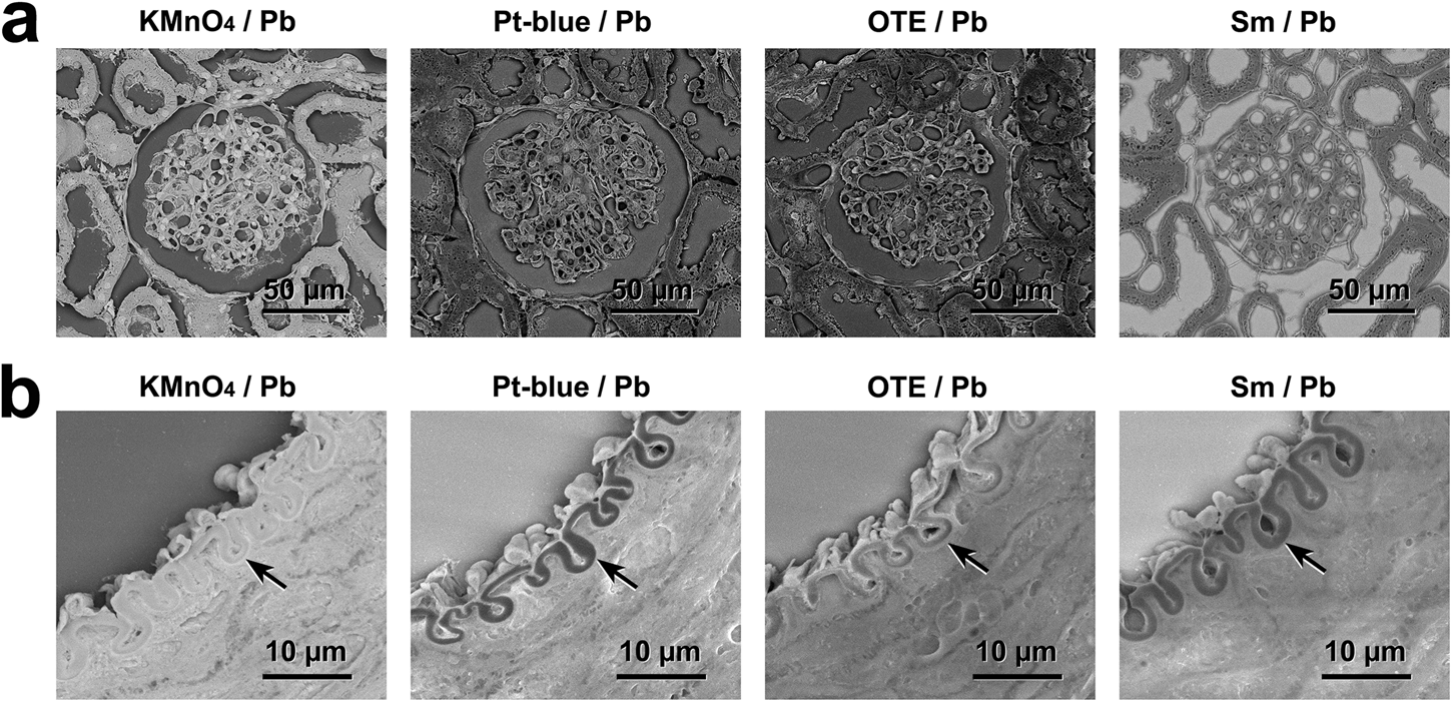
Comparison of PS-LvSEM imaging after KMnO_4_/Pb, Pt-blue/Pb, OTE/Pb and Sm/Pb metal staining. **a** Renal corpuscle and uriniferous tubules. **b** Muscular artery in the kidney. Arrows indicate internal elastic lamina. KMnO_4_/Pb: 0.2% KMnO_4_ for 5 min followed by Pb staining for 3 min. Pt-blue/Pb: Pt-blue (pH 9) for 15 min followed by Pb staining for 3 min. OTE/Pb: 0.2% OTE in 0.1 M PB for 20 min followed by Pb staining for 3 min. Sm/Pb: 2.5% samarium triacetate for 20 min followed by Pb staining for 3 min. Note the highest contrast by KMnO_4_/Pb metal staining in comparison with conventional uranium-free Pt-blue/Pb, OTE/Pb and Sm/Pb metal staining.

Comparative LvSEM images clearly demonstrated the highest contrast by KMnO_4_/Pb metal staining compared with conventional uranium-free Pt-blue/Pb, OTE/Pb, and Sm/Pb metal staining (Fig. 6). In contrast to KMnO_4_/Pb metal staining, these conventional uranium-free metal staining methods provided dark areas in the internal elastic lamina of the muscular artery (Fig. 6b) as shown in Ur/Pb metal staining (Fig. 4f).

### Application of multiscale LvSEM imaging of cell/tissue architectures by KMnO_4_/Pb metal staining

**Fig. 7:**
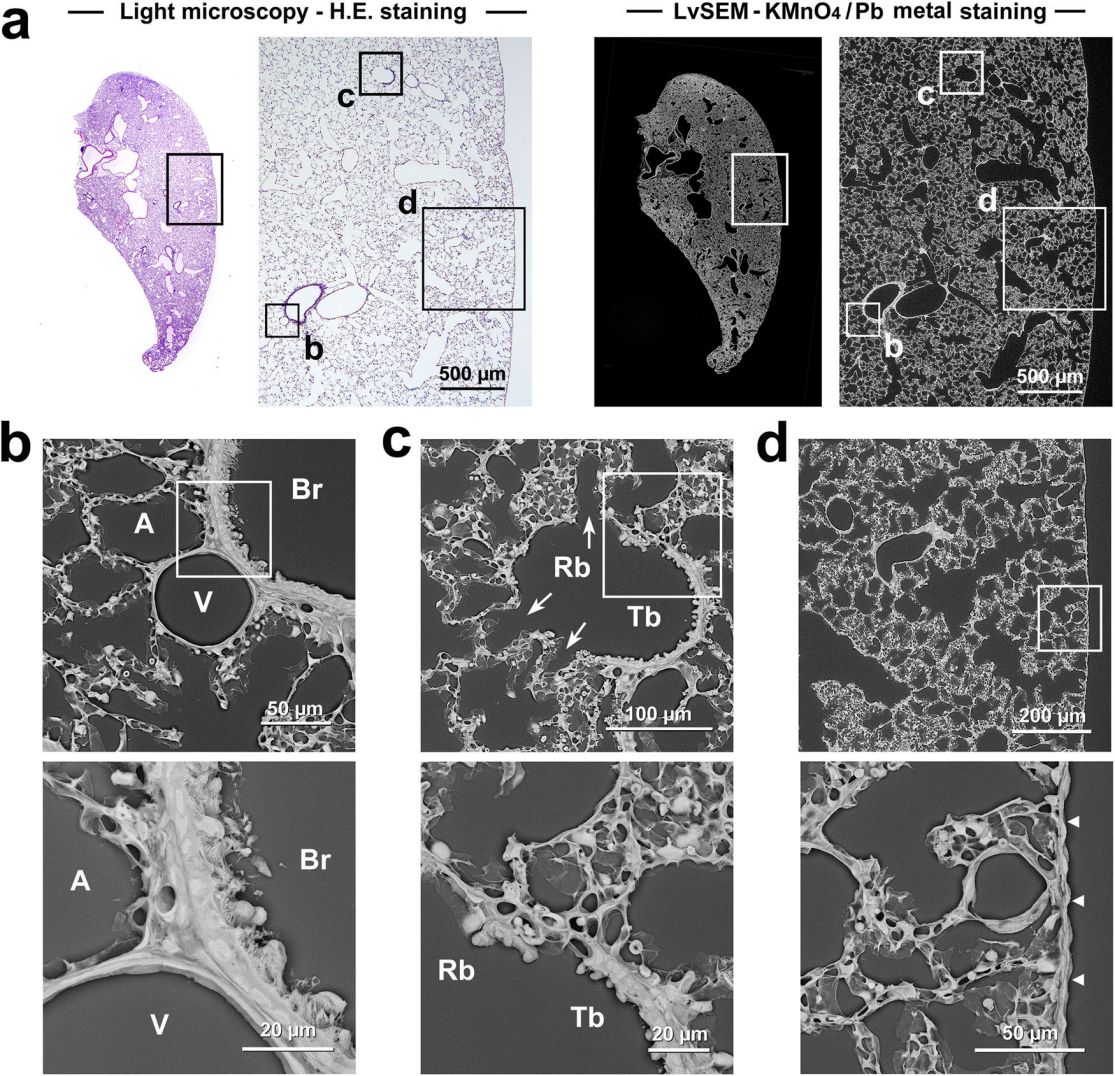
Correlative light microscopy and PS-LvSEM images of rat lungs after KMnO_4_/Pb metal staining. **a** Overview of rat lungs by correlative light microscopy and PS-LvSEM imaging. **b**–**d** Representative PS-LvSEM images after KMnO_4_/Pb metal staining corresponding to the light micrographs. **b** Distinct ultrastructure of the ciliated cuboidal epithelium of the bronchiole (Br), simple squamous endothelium of the blood vessel (V), and thin capillary vessels surrounding the alveoli (A). **c** Transition from the terminal bronchiole (Tb), which consists of nonciliated cuboidal epithelium, to the respiratory bronchiole (Rb), which branches to the alveoli via openings (arrows). **d** Spongy structure of the lung parenchyma consisting of alveoli covered by the visceral pleura (arrowheads).

The optimised KMnO_4_/Pb metal staining procedure provided correlative light microscopy and PS-LvSEM images of rat lung paraffin sections (Fig. 7). Thus, high-power views of the ciliated cuboidal epithelium of the bronchiole, simple squamous endothelium of the blood vessel, and capillary vessels surrounding the alveoli were taken as representative ultrastructures (Fig. 7b). Beyond the light microscopic level, the cell/tissue ultrastructure was also visualised in the transition from the terminal bronchiole consisting of nonciliated cuboidal epithelium to the respiratory bronchiole branching into the alveoli (Fig. 7c), and the spongy structure of the lung parenchyma was covered by the visceral pleura (Fig. 7d).

**Fig. 8:**
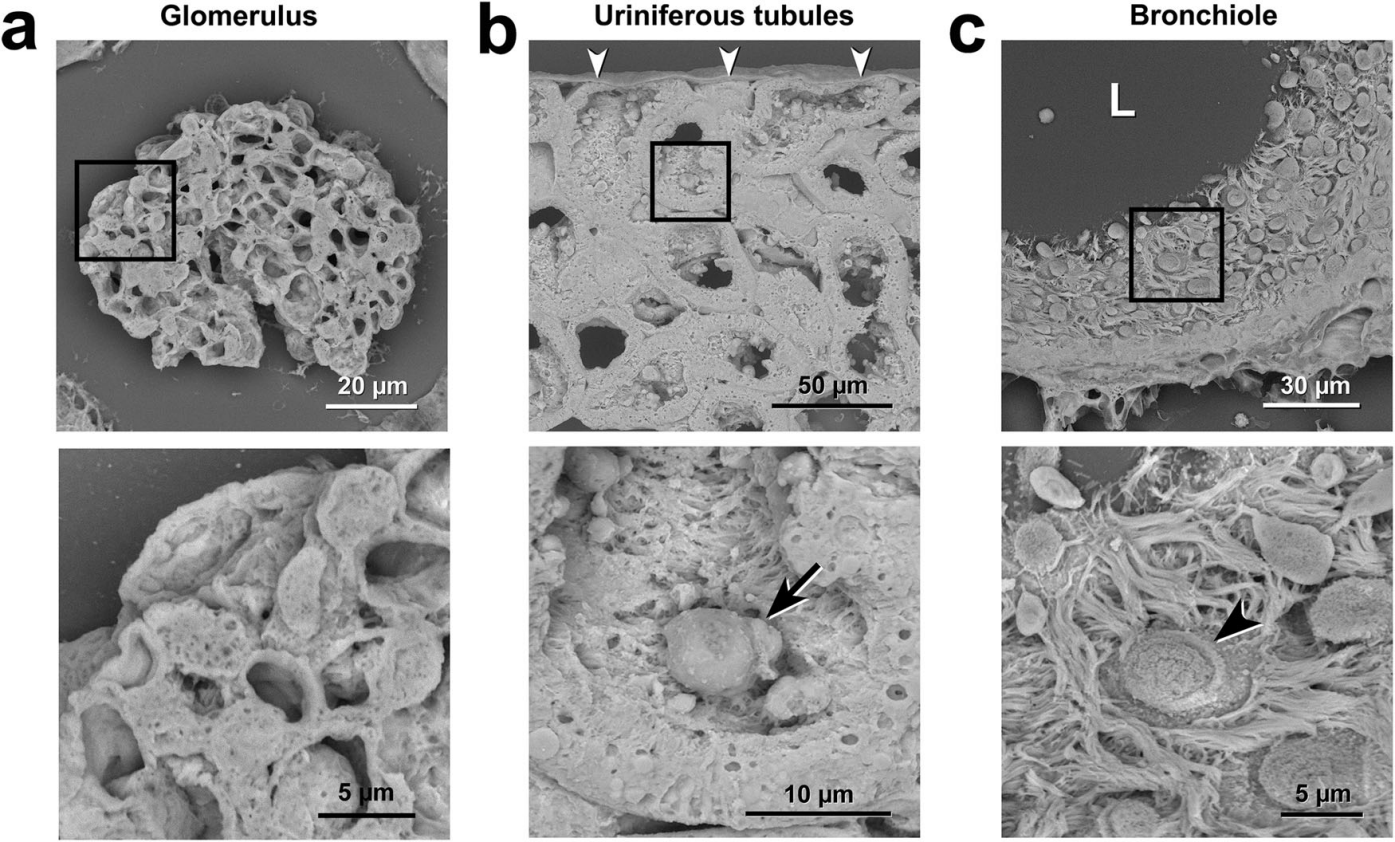
Three-dimensional cell/tissue architectures in 20-µm-thick sectioned organs. Representative Thick PS-LvSEM micrographs of 20-µm-thick sections after KMnO_4_/Pb metal staining. **a** Glomerulus. Overview (upper) of the glomerulus within the renal corpuscle and high-power view (lower) of the podocytes covering the glomerular capillaries with their processes. **b** Uriniferous tubules. Overview (upper) of the uriniferous tubules beneath the renal capsules (arrowheads) and high-power view (lower) of the microvilli and the exfoliated epithelial cells in the lumen (lower, arrow). **c** Bronchioles in the lung. Overview (upper) of the bronchioles consisting of ciliated epithelium and high-power view (lower) of the exocrine cells (arrowhead) among the ciliated cells.

We applied KMnO_4_/Pb metal staining to Thick PS-LvSEM for three-dimensional imaging of cell/tissue architectures (Fig. 8). In the renal corpuscle, the podocytes were observed to cover the glomerular capillaries with their processes (Fig. 8a). In the uriniferous tubules, the fine structures of the microvilli and exfoliated epithelial cells were clearly observed in the lumen of the proximal convoluted tubes (Fig. 8b). In the lung bronchioles, Thick PS-LvSEM imaging revealed that the cilia protruded from the respiratory epithelium (Fig. 8c). Observation of the 20 µm-thick section facilitated distinctive perception of the face-side (instead of sectioned) images of the epithelium (Fig. 8b, c), which is rarely noted within thin sections.

### Fine structural preservation by fixation with conventional 10% formalin (4% paraformaldehyde) without glutaraldehyde

**Fig. 9:**
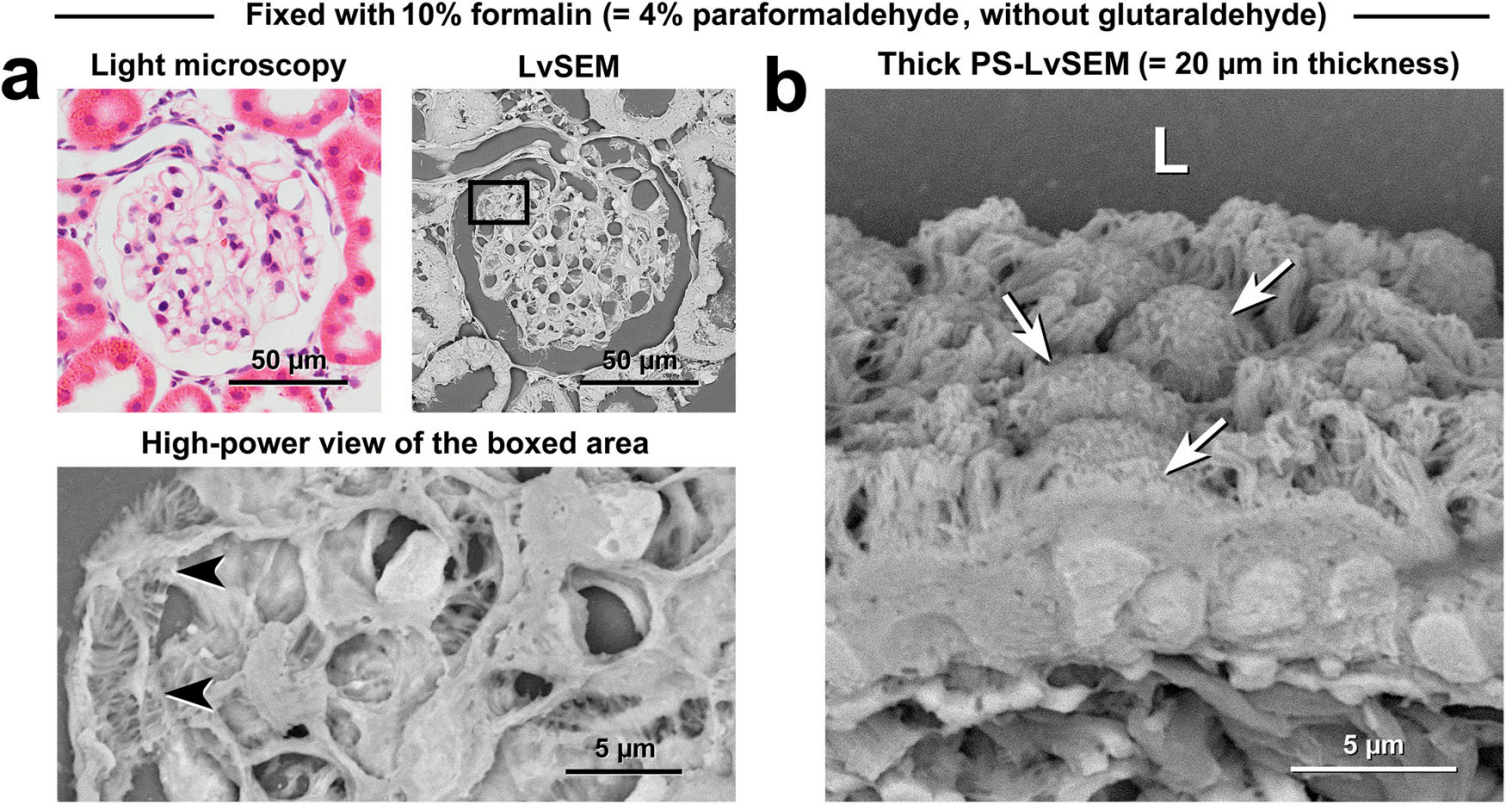
Fine structural preservation by fixation with conventional 10% formalin (4% paraformaldehyde) without glutaraldehyde. **a** Correlative light microscopy and PS-LvSEM images of the renal corpuscle. The high-power view shows the processes of podocytes (arrowhead) in the glomerulus. **b** Thick PS-LvSEM micrographs of the bronchioles. Note the three-dimensional cell/tissue architectures in 20-µm-thick sections consisting of ciliated cuboidal cells and exocrine cells (arrows).

Figure 9 shows representative fine structures preserved by fixation with conventional 10% formalin (4% paraformaldehyde) without glutaraldehyde. Multiscale correlative light microscopy and PS-LvSEM imaging demonstrated well-preserved cell/tissue architectures from the overview of the renal corpuscle to the ultrastructure of podocytes and their processes within the glomerulus (Fig. 9a). Thick PS-LvSEM demonstrated the three-dimensional architectures of ciliated cuboidal cells and exocrine cells in the bronchioles (Fig. 9b).

## Discussion

High-resolution imaging by electron microscopy is the best way to elucidate complex cell/tissue architectures^1,2^. SEM is a type of electron microscopy that produces magnified images of a specimen by scanning its surface with a focused electron beam to create a high-resolution image. The reflected or backscattered electrons originate from the specimen by elastic scattering interactions with the specimen atoms. Thus far, combined staining with uranyl acetate^16^ followed by lead citrate^22^ has long been applied for the high-contrast biomedical electron microscopic imaging of cell/tissue ultrastructures because heavy elements backscatter electrons more strongly than light elements. However, the use of radioactive uranyl compounds has been restricted by law, which hampers the universal application of electron microscopy. Here, uranium-free KMnO_4_/Pb metal staining allows multiscale imaging of cell/tissue ultrastructures in paraffin sections. The obtained image contrast is equivalent to that of Ur/Pb metal staining, making this method sufficient for ultrastructural observation via LvSEM.

In electron microscopy, KMnO_4_ has been used for fixation^23^ to increase the electron density or for staining^24,25^ to enhance the image contrast. We previously applied KMnO_4_ oxidation to visualise the zymogenic contents in the gastric gland^26^ and for contrast enhancement^27^ combined with Ur/Pb metal staining for Lowicryl K4M ultrathin sections. The general enhancement observed in these previous studies encouraged us to further apply KMnO_4_ oxidation of paraffin sections. In contrast to the conventional uranium-free metal staining methods, KMnO_4_/Pb metal staining successfully yielded a sufficient BSE signal to entirely visualise the cell/tissue ultrastructure. The image contrast of the obtained images was equivalent to that of Ur/Pb staining and was sufficient for ultrastructural observation, clearly demonstrating the presence of podocytes and their processes in the renal corpuscle. Control treatment with reduced KMnO_4_ suggested the importance of oxidation to increase the BSE signal intensity for subsequent Pb deposition.

Elemental analysis was verified using KMnO_4_/Pb and Ur/Pb metal staining by comparing the heavy metal deposition and the resulting BSE signal intensity. The present analysis revealed that the determinant of BSE signal intensity was the amount of Pb deposited during both KMnO_4_/Pb and Ur/Pb metal staining. Interestingly, KMnO_4_/Pb metal staining exhibited intense BSE signals in the inner elastic lamina of the muscular artery and the interterritorial matrix of the elastic cartilage, which was distinct from the Ur/Pb metal staining. These findings implied the distinct histochemical properties of KMnO_4_ oxidation and Ur staining, although both treatments enhanced Pb deposition. The interterritorial matrix is occupied by a network of anastomosing elastic fibres consisting of elastin^28^ that appears electron lucent under a transmission electron microscope by Ur/Pb metal staining^28–30^. This low affinity of elastin for Ur/Pb metal staining is consistent with the low BSE signal in the interterritorial matrix. It has been assumed that alterations in proteins due to KMnO_4_ oxidation yield anionic binding sites for lead citrate^31–33^. Further histochemical investigations are needed to clarify the precise mechanism of KMnO_4_ oxidation and subsequent increase in Pb deposition, aiming to determine the optimal heavy metal for enhancing the BSE signal in LvSEM.

By exploiting low-vacuum conditions, PS-LvSEM provides multiscale imaging of nonconductive biomedical specimens from extensive cell/tissue architectures to intensive subcellular ultrastructures that are invisible under a light microscope. The camera navigation system equipped with compact LvSEM promotes CLEM imaging to determine the orientation of complex cell/tissue architectures and identify ultrastructural correlations through an operation screen. From the centimetre to the nanometre scale, multiscale imaging will increase the scientific reliability of electron micrographs corresponding to light micrographs.

BSE imaging has been widely applied to high-resolution imaging techniques, such as volume electron microscopy^34–36^, which are used in biomedical research to reveal the 3D structure of cells, tissues, and small model organisms at nanometre resolution. However, these techniques require time-consuming sample preparation and image acquisition methods. In addition, their maximum specimen widths are restricted to a few millimetres, which is much smaller than the centimetre scale for PS-LvSEM. The application of KMnO_4_/Pb metal staining to Thick PS-LvSEM provided characteristic three-dimensional images of complex cell/tissue architectures at large depths by means of very narrow electron beam scanning over thick paraffin sections^10^. In LvSEM, a lower accelerating voltage is sufficient to capture the surface structure because a higher accelerating voltage results in a greater depth of beam penetration missing the BSE signal from the surface^6,37,38^; however, a lower accelerating voltage yields a lower BSE signal intensity as a trade-off. Based on the present findings, the use of a lower accelerating voltage (5–10 kV) is recommended for identifying the subcellular ultrastructure at higher magnification, and a higher accelerating voltage (15–20 kV) is recommended for observing the cell/tissue architecture at lower magnification.

Recent advances in biomedical research, such as the production of regenerated three-dimensional organoids (e.g. kidney^39^, lung^40^, thyroid^41^ and cartilage^42^, whose cell/tissue architectures were shown in this study) from induced pluripotent stem cells and the morphological changes induced by CRISPR/Cas9-mediated genome editing^43^, require the ultrastructural examination of a phenotype induced by particular gene and/or molecule expression. Further application of Thick PS-LvSEM is highly anticipated to accelerate these biomedical challenges by revealing the three-dimensional cell/tissue architectures because uranium-free KMnO_4_/Pb metal staining enables the universal operation of LvSEM in biomedical research institutes.

It is generally thought that electron microscopy is a troublesome technique requiring skilful and time-consuming sample preparation as well as complicated operation of the electron microscope, but sample preparation for KMnO_4_/Pb metal staining requires no special equipment or techniques. For light microscopic examinations, paraffin wax is widely used in histology and pathology because it is inexpensive and easy to use for sectioning. The present uranium-free KMnO_4_/Pb metal staining method allows for the universal use of PS-LvSEM in hospitals as a tool for multidimensional microscopy for histopathological diagnosis^44^. It is also important to note that the paraffin-embedded samples can be stored for decades and that their use in PS-LvSEM with KMnO_4_/Pb metal staining enables a retrospective investigation of valuable previously acquired samples^45^. Although most of the specimens were fixed with glutaraldehyde in this study for superior ultrastructural preservation, it should be kept in mind that the present method is applicable to conventional 10% formalin (4% paraformaldehyde)-fixed specimens for ultrastructural observation, as shown here and in previous reports^8–10,14,15^. Importantly, without glutaraldehyde, the specimens can be applied to CLEM imaging that combines the molecular specificity of fluorescence microscopy and the high spatial resolution of electron microscopy by PS-LvSEM with KMnO_4_/Pb metal staining.

In conclusion, we developed a uranium-free KMnO_4_/Pb metal staining method for multiscale imaging of cell/tissue ultrastructures by LvSEM and described the precise procedures while presenting representative electron micrographs. The present user-friendly method will enable the universal operation of LvSEM and create a new approach for biomedical electron microscopy, bridging the gap between light and electron microscopy.

## Methods

### Preparation of paraffin sections

Ten-week-old male Wistar rats (Kyudo, Kumamoto, Japan) were deeply anaesthetised via isoflurane inhalation and then perfused with 10% formalin (4% paraformaldehyde) or a mixture of 2% paraformaldehyde and 2.5% glutaraldehyde in 0.1 M phosphate buffer (PB: pH 7.4) through the left ventricle of the heart. The kidney with the renal artery and ureter, lung, trachea with the thyroid gland, and auricle were excised and further fixed by immersion in the above fixative for 2 h at room temperature (RT) (Fig. 1a). After washing in running tap water for 2 h, the organs were dehydrated in a graded series of ethanol (50, 70, 80, 90 and 100%) and cleared by xylene for 2 h through an automatic tissue processor (TP 1020, Leica Microsystems, Wetzlar, Germany) to be embedded in paraffin (melting point 54–56 °C: Wako Pure Chemical Industries, Osaka, Japan) using a heated paraffin embedding station (HistoCore Arcadia H, Leica Microsystems GmbH). Then, thin 5-μm sections or thick 20-μm sections were cut using a sliding microtome (Fig. 1b). The sections were floated in a water bath (PS-125WH, Sakura Finetek Japan, Tokyo, Japan) at 40 °C to be mounted onto New Silane II-coated microscope slides (Muto Pure Chemical, Tokyo, Japan) and extended on a slide warmer (PS-53, Sakura Finetek Japan). After drying in an incubator at 37 °C overnight, the sections were deparaffinized in xylene and rehydrated in a series of ethanol (100, 90, 70 and 50%) and running tap water (5 min each).

### Correlating light microscopy and LvSEM imaging

Deparaffinized and rehydrated sections were stained with Mayer’s haematoxylin for 5 min and exposed to running tap water for at least 1 h to develop the colour. Next, the sections were stained with eosin diluted in 60% ethanol for 3 min and then rinsed in a graded series of ethanol (80, 90 and 100%) for dehydration. After clearing in xylene, the sections were mounted with NEW MX (Matsunami Glass, Osaka, Japan) and covered with a NEO Micro cover glass (size 24 × 50 mm, thickness No. 1 = 0.13–0.17 mm: Matsunami Glass). After being observed under a light microscope (BX51, Olympus, Tokyo, Japan) equipped with a digital camera (DP72, Olympus) (Fig. 1c), the microscope slides were incubated in xylene for 18–24 h at room temperature to remove the coverslips (Fig. 1d). The sections were rehydrated with a series of ethanol (100, 90 and 70%) and distilled water (5 min each) and then subjected to metal staining for LvSEM imaging.

### KMnO_4_/Pb metal staining and conventional metal staining

The sections were treated with 0.2% potassium permanganate (KMnO_4_) for 5 min. After washing with distilled water, the sections were treated with Reynolds’ lead citrate solution (Pb) for 3 min (KMnO_4_/Pb metal staining) (Fig. 1e). After washing and drying, the sections were ready for observation. The optimal protocol was determined by the coordination of KMnO_4_ concentration (0.1, 0.2 or 0.5%) and KMnO_4_ treatment duration (1, 3, 5 or 10 min) in comparison with conventional metal staining with 1% uranyl acetate in 70% methanol for 5 min followed by Pb for 3 min (Ur/Pb metal staining). As controls, sections were treated with 0.2% KMnO_4_ for 5 min alone, Pb for 3 min alone, or Pb for 3 min followed by 0.2% KMnO_4_ for 5 min. Oxidation dependence was examined with reduced KMnO_4_ (prepared by adding 0.1% hydrogen peroxide until the solution became translucent) for 5 min or 1% osmium tetroxide in 0.1 M PB for 5 min. As a comparison, sections were treated with platinum blue (Nisshin EM Co. Ltd., Tokyo, adjusted to pH 9 by adding a small volume of ammonia solution) for 15 min (Pt-blue/Pb metal staining)^17,21^, 0.2% oolong tea extract (Nisshin EM Co. Ltd., Tokyo) in 0.1 M PB for 20 min (OTE/Pb metal staining)^18^, or 2.5% samarium triacetate (Wako Pure Chemical Industries, Osaka, Japan)^19,20^ for 20 min (Sm/Pb metal staining), followed by Pb for 3 min.

### LvSEM

The microscope slides were subjected to LvSEM (TM4000Plus II, Hitachi High-Tech, Tokyo, Japan) (Fig. 1f) and then observed under electron beam accelerating voltages of 5, 10, 15V or 20 kV. Whole-section montage images were obtained by the semiautomatic capture of tilling electron micrographs (Supplementary Fig. S1). For histogram analysis, 12 electron micrographs were collected at random from the renal cortex (Supplementary Fig. S3). After tilling the collected images, grayscale histograms were obtained by using NIH ImageJ software (version 1.53 s). Elemental analysis was performed by using an energy-dispersive X-ray (EDX) detector equipped with LvSEM.

All animal procedures were carried out under protocols approved by the University of Miyazaki Animal Research Committee in accordance with international guiding principles for biomedical research involving animals.

## Supplementary information

Supplementary information

## Data Availability

No datasets were generated or analysed during the current study.
