# Supplementary material for: KMnO4/Pb staining allows uranium free imaging of tissue architectures in low vacuum scanning electron microscopy
Source: Npj Imaging. 2024 Oct 1;2:40. doi: 10.1038/s44303-024-00045-z (PMC12118665; doi:10.1038/s44303-024-00045-z)
Supplement: Supplementary file 1 — Supplementary information [file 44303_2024_45_MOESM1_ESM.pdf]

Supplementary information

KMnO4/Pb staining allows uranium free imaging of tissue architectures in low vacuum scanning electron microscopy

Akira Sawaguchi[1, *], Takeshi Kamimura[2], Kyoko Kitagawa[1], Yoko Nagashima[1], Nobuyasu Takahashi[1]

1. Division of Ultrastructural Cell Biology, Department of Anatomy, Faculty of Medicine, University of Miyazaki, Miyazaki 889-1692, Japan.

2. Hitachi High-Tech Corporation, Tokyo 105-6409, Japan.

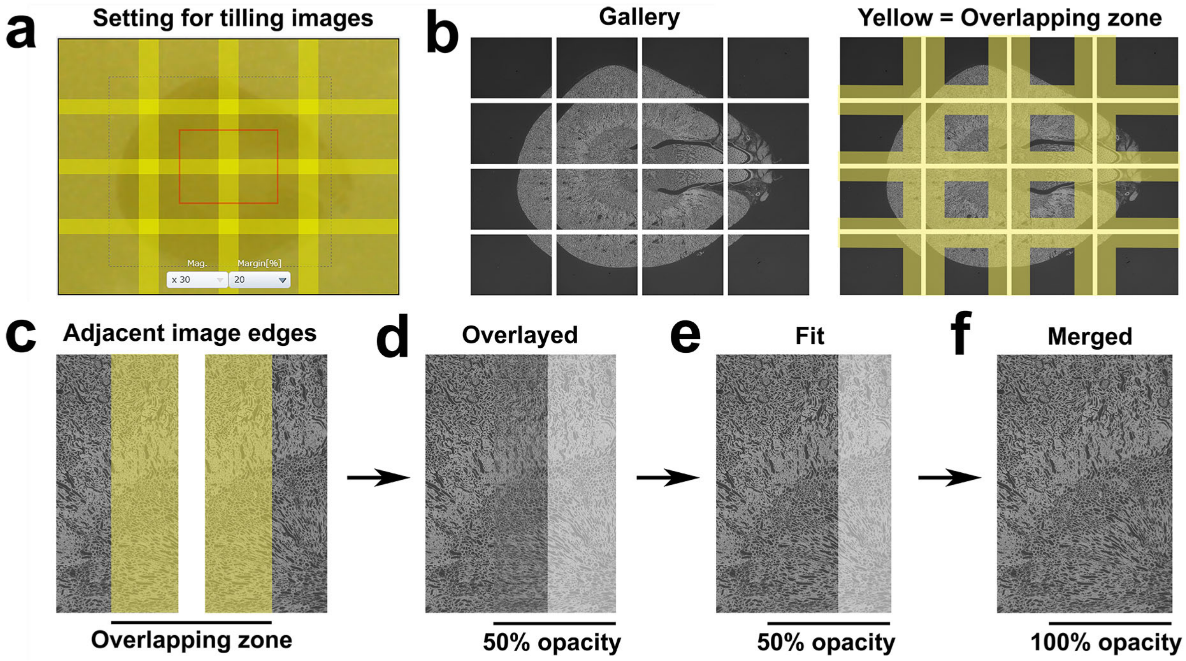

Supplementary Figure 1

Semiautomatic capture and processing of a whole-section montage image.

(**a**) Setting for the semiautomatic capture of tilling electron micrographs. The bright yellow lines indicate the overlapping zone. (**b**) Gallery of the collected images. (**c-f**) Processing of the overlayed fit and merging to obtain the final montage image.

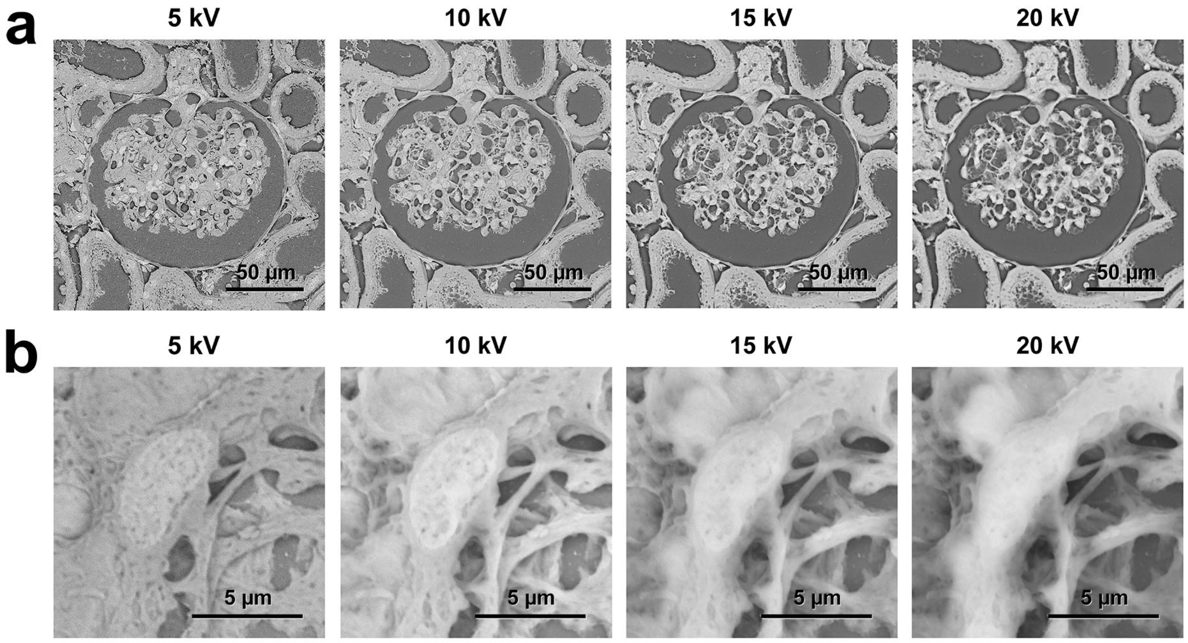

Supplementary Figure 2

Tuning the acceleration voltage for PS-LvSEM imaging by $KMnO_4$/Pb metal staining.

Representative PS-LvSEM images at acceleration voltages of 5, 10, 15, and 20 kV. (**a**) Low-power view of the renal corpuscle. (**b**) High-power views of podocytes in the glomerulus. Note the trade-off between the low contrast revealing surface undulations at 5 kV and the high contrast missing surface undulations at 20 kV.

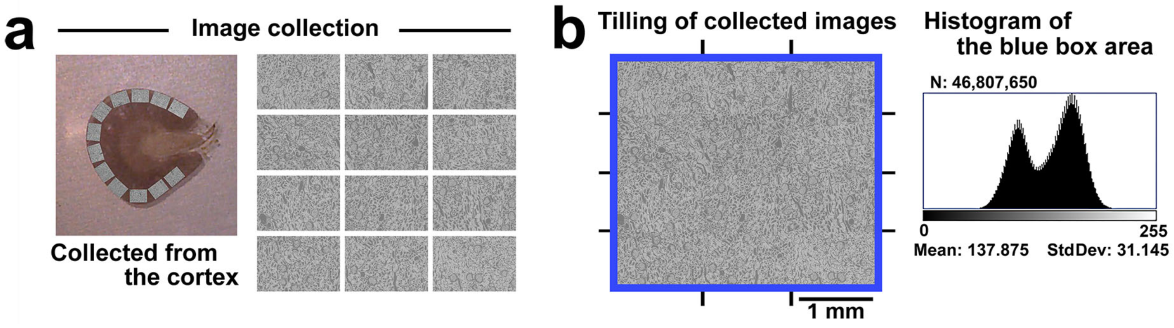

Supplementary Figure 3

Collection and tilling electron micrographs for histogram analysis.

(**a**) Collection of 12 random electron micrographs from the renal cortex. (**b**) Tilling of the collected images for histogram analysis. N: total number of pixels; Mean: mean grey level; StdDev: standard deviation.

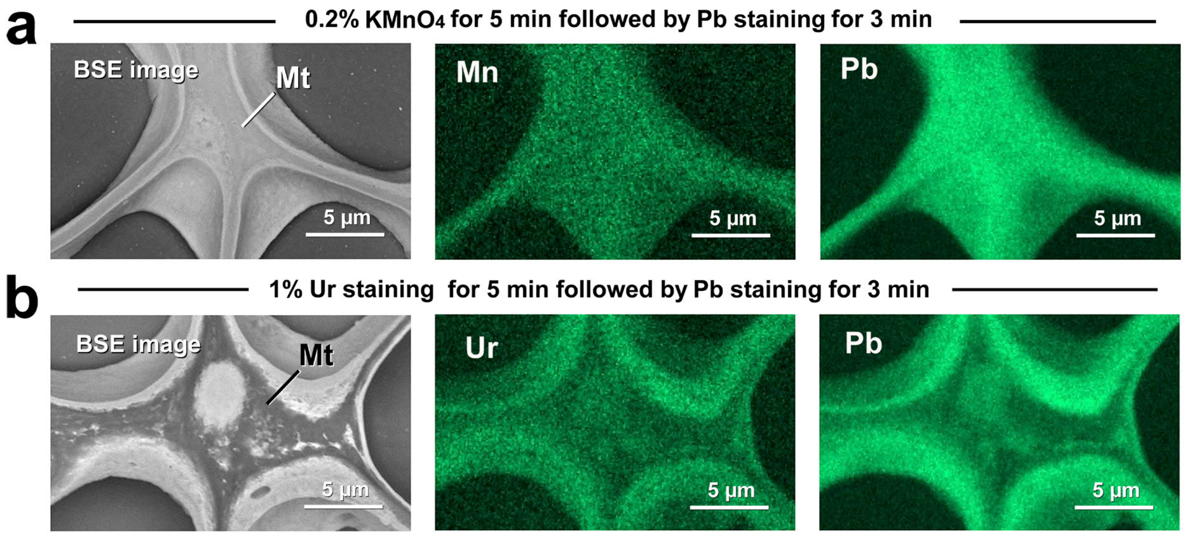

Supplementary Figure 4

Elemental analysis of the metal distribution after $KMnO_4$/Pb and Ur/Pb metal staining.

Elemental mapping showing the distributions of manganese (Mn), lead (Pb), and uranium (Ur) after treatment with 0.2% $KMnO_4$ for 5 min, followed by Pb staining for 3 min (**a**) and after treatment with 1% uranyl acetate for 5 min, followed by Pb staining for 3 min (**b**). The intense Mn and Pb distributions were consistent with the high-contrast PS-LvSEM image of the interterritorial matrix (Mt) obtained by $KMnO_4$/Pb metal staining.
